# CD44 acts as a coreceptor for cell-specific enhancement of signaling and regulatory T cell induction by TGM1, a parasite TGF-β mimic

**DOI:** 10.1073/pnas.2302370120

**Published:** 2023-08-17

**Authors:** Maarten van Dinther, Kyle T. Cunningham, Shashi Prakash Singh, Madeleine P. J. White, Tiffany Campion, Claire Ciancia, Peter A. van Veelen, Arnoud H. de Ru, Román González-Prieto, Ananya Mukundan, Chang-Hyeock Byeon, Sophia R. Staggers, Cynthia S. Hinck, Andrew P. Hinck, Peter ten Dijke, Rick M. Maizels

**Affiliations:** ^a^Oncode Institute and Department of Cell and Chemical Biology, Leiden University Medical Center, Leiden 2300 RC, The Netherlands; ^b^Wellcome Centre for Integrative Parasitology, School of Infection and Immunity, University of Glasgow, Glasgow G12 8TA, United Kingdom; ^c^Center for Proteomics and Metabolomics, Leiden University Medical Center, Leiden 2333 ZC, The Netherlands; ^d^Andalusian Center for Molecular Biology and Regenerative Medicine, Universidad de Sevilla - CSIC - Universidad Pablo de Olavide, 41092 Sevilla, Spain; ^e^Department of Cell Biology, Faculty of Biology, University of Sevilla, 41013 Sevilla, Spain; ^f^Department of Structural Biology, University of Pittsburgh School of Medicine, Pittsburgh, PA 15260

**Keywords:** complement control protein, convergent evolution, fibroblast, *Heligmosomoides polygyrus*, T lymphocyte

## Abstract

Long-lived parasites evade host immunity through highly evolved molecular strategies. The murine intestinal helminth, *Heligmosomoides polygyrus*, down-modulates the host immune system through release of an immunosuppressive TGF-β mimic, TGM1, which is a divergent member of the CCP (Sushi) protein family. TGM1 comprises 5 domains, of which domains 1-3 (D1/2/3) bind mammalian TGF-β receptors, acting on T cells to induce Foxp3^+^ regulatory T cells; however, the roles of domains 4 and 5 (D4/5) remain unknown. We noted that truncated TGM1, lacking D4/5, showed reduced potency. Combination of D1/2/3 and D4/5 as separate proteins did not alter potency, suggesting that a physical linkage is required and that these domains do not deliver an independent signal. Coprecipitation from cells treated with biotinylated D4/5, followed by mass spectrometry, identified the cell surface protein CD44 as a coreceptor for TGM1. Both full-length and D4/5 bound strongly to a range of primary cells and cell lines, to a greater degree than D1/2/3 alone, although some cell lines did not respond to TGM1. Ectopic expression of CD44 in nonresponding cells conferred responsiveness, while genetic depletion of CD44 abolished enhancement by D4/5 and ablated the ability of full-length TGM1 to bind to cell surfaces. Moreover, CD44-deficient T cells showed attenuated induction of Foxp3 by full-length TGM1, to levels similar to those induced by D1/2/3. Hence, a parasite protein known to bind two host cytokine receptor subunits has evolved a third receptor specificity, which serves to raise the avidity and cell type–specific potency of TGF-β signaling in mammalian cells.

## Significance

Pathogens target key immune pathways for their survival. The TGF-β mimic (TGM1) secreted by *Heligmosomoides polygyrus* binds host TGF-β receptors to trigger immune suppressive pathways, utilizing different domains to bind each of the receptor subunits. We now show additional domains of TGM1 bind CD44, which both maximizes its signaling potency and delivers its signal in a cell type–specific manner. Thus, multiple modules of TGM1 represent a unique and effective mode of selective immune modulation by a highly evolved parasite.

Many parasites maintain long-term infections by down-modulation of host immunity through the release of soluble extracellular factors that modulate or suppress key immune pathways (1–4). We recently described a functional mimic of the immunosuppressive cytokine, transforming growth factor (TGF)-β, with antiinflammatory properties, that is released by the murine intestinal helminth, *Heligmosomoides polygyrus* (5). This mimic, named TGM1, has no structural similarity to the TGF-β protein but effectively activates TGF-β receptor signaling in murine and human lymphocytes resulting in, for example, the induction of immunosuppressive regulatory T cells expressing the transcription factor Foxp3 (6, 7). In vivo, TGM1 is able to moderate a range of inflammatory conditions, including allograft rejection (5), airway allergy (8), and intestinal colitis induced by dextran sodium sulfate or by transfer of effector T cells (9, 10).

TGM1 is a secreted 422-aa protein comprising five homologous domains, each with structural similarity to the CCP (Sushi) protein family (11). While mammalian TGF-β is proteolytically processed from a secreted latent preprotein into a biologically active 112-aa product that is found in a covalent dimeric form, TGM1 is biologically active as a full-length monomeric protein. Both secreted proteins signal on mammalian cells through the same cell surface transmembrane receptor assembly, i.e., TGF-β type I receptor (TβRI) and TGF-β type II receptor (TβRII) that are endowed with serine/threonine kinase activity (5). Notably, while TGF-β binds TβRI only once TGF-β is complexed to TβRII (12), TGM1 independently binds both TβR subunits, with domains 1 and 2 binding TβRI and domain 3 binding TβRII (11). Thus, compared to TGF-β, TGM1 employs a distinct molecular mechanism to assemble the TβRII/TβRI complex and activate the same intracellular SMAD transcriptional effectors. Within TGM1, domains 1 to 3 (D1/2/3) are necessary and sufficient for signaling (13). In contrast to these observations, however, a role for domains 4 and 5 (D4/5) has yet to be defined at either the molecular or cellular level.

Besides TβRI and TβRII, TGF-β utilizes the coreceptor betaglycan [also termed TGF-β type III receptor (TβRIII)] to present ligand to the signaling receptors (14). As different cell types have different coreceptor expression, they appear to have an important role in determining cell specificity by facilitating TGF-β signaling responses. In addition, coreceptors may participate in other ways, for example, by recruiting proteins to signaling receptors or regulating their intracellular trafficking (15).

In this report, we address the question of whether D4/5 contributes to the biological function of the parasite TGF-β mimic; we show that these domains directly contact a distinct cell surface protein, identified as CD44, a widely variable expressed cell surface protein with roles ranging from cell adhesion to signaling and differentiation (16). We show that in the absence of D4/5, TGM1 no longer interacts with CD44, and its ability to trigger TGF-β signaling is sharply attenuated, but not abolished. We also find that TGM1, derived from a mouse-adapted parasite, interacts more strongly with murine, compared to human, CD44. Our findings highlight the remarkable ability of TGM1 protein to independently ligate three separate receptor entities on the host cell surface and uncover a coreceptor function for CD44, with binding of TGM1 D4/5 to CD44, thereby conferring enhanced avidity and preferential cell targeting by the parasite TGM1 immunomodulator.

## Results

### TGM1 Domains 4 and 5 Maximize the Potency of the TGF-β Receptor Binding Domains 1–3.

**Fig. 1.**
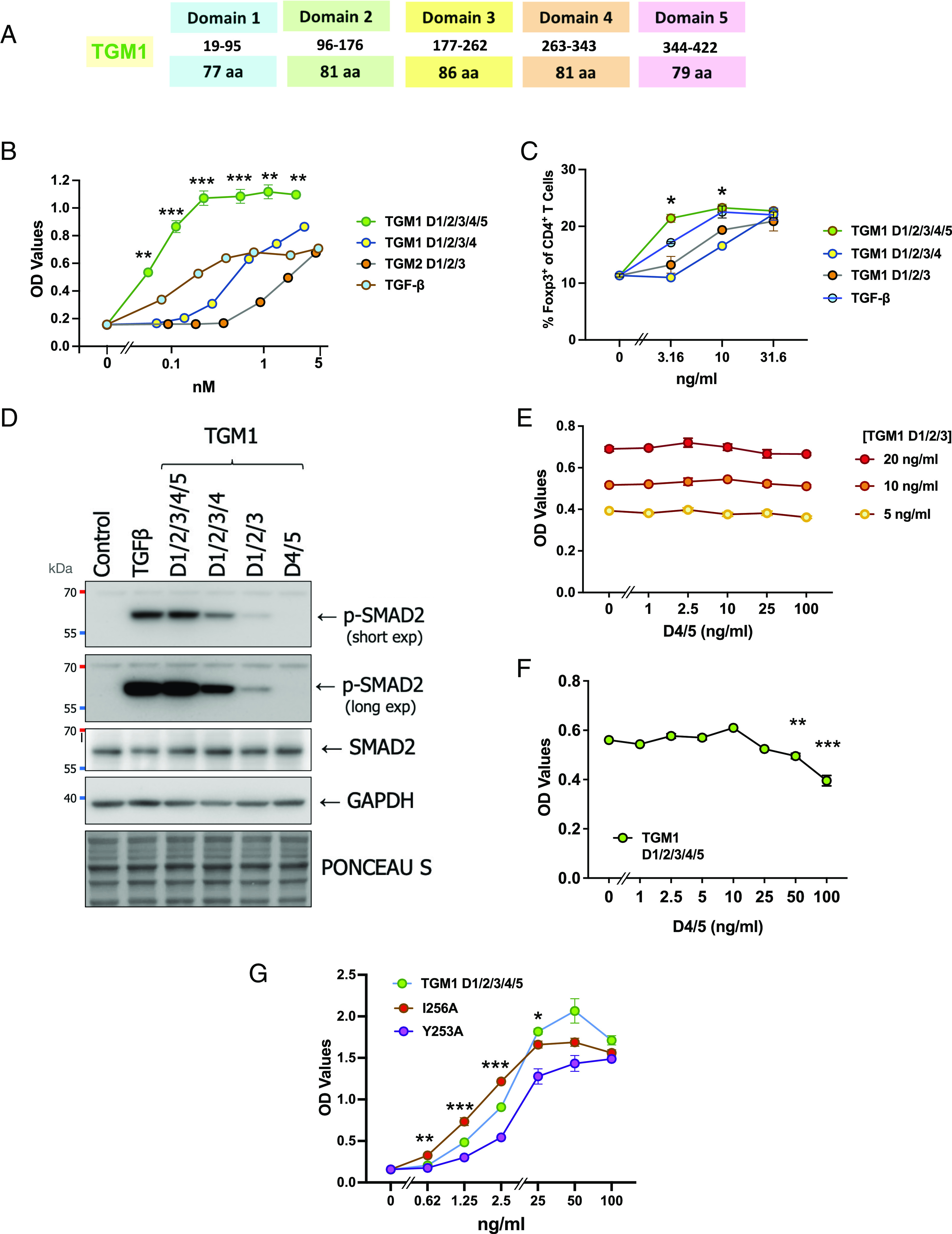
Domains 4 and 5 of TGM1 are required for full potency of signaling. (*A*) Schematic of 5-domain structure of *H. polygyrus* TGM1, indicating sizes in amino acids (aa). An 18-aa N-terminal signal peptide is not depicted. Note that TGM1 is biologically active as a full-length protein once its signal peptide has been cleaved. (*B*) Loss of D5 or D4/5 reduces the activity of TGM1 in the MFB-F11 fibroblast reporter bioassay to below that of full-length (D1/2/3/4/5) TGM1 or TGF-β. Alkaline phosphatase secreted by reporter cells was measured at 24 h by ELISA assay. Statistical comparisons between D1/2/3/4/5 and D1/2/3 are shown, **P < 0.01, ***P < 0.001 by unpaired *t* test corrected for multiple comparisons. (*C*) Loss of D5 or D4/5 reduces potency of Foxp3 induction in primary splenic CD4^+^ T cells incubated for 4 d with anti-CD3 and interleukin (IL)-2. Statistical comparisons between D1/2/3/4/5 and D1/2/3 are shown, *P < 0.05, by unpaired *t* test corrected for multiple comparisons. (*D*) SMAD2 phosphorylation measured by Western blot analysis of NM18 cells incubated for 1 h with 1 ng/mL TGF-β or 10 ng/mL TGM1 full-length (D1/2/3/4/5), or truncated TGM1 proteins containing the indicated domains. Mol.wt markers are shown in kDa on the left-hand side. (*E*) Addition of TGM1 D4/5 separately from D1/2/3 does not restore TGM1 full length response in MFB-F11 cells. (*F*) TGM1 D4/5 at doses above 50 ng/mL inhibits full length (D1/2/3/4/5) response in MFB-F11 cells. (*G*) Y253A mutation in full-length (D1/2/3/4/5) TGM1 retains biological activity when tested on MFB-F11 cells, albeit at reduced potency. Statistical comparisons between wild-type and Y253A mutant are shown, *P < 0.05. **P < 0.01, ***P < 0.001 by unpaired *t* test corrected for multiple comparisons.

TGM1 is a secreted helminth parasite-derived protein that activates mammalian TGF-β signaling despite bearing no sequence similarity or structural homology to TGF-β family members (5) and consists of five homologous but nonidentical domains of around 80aa (Fig. 1*A*). Monomeric TGM1 binds both subunits of the mammalian TGFβR, with domains 1 and 2 (D1/2) binding TβRI with high affinity (~60 nM) and domain 3 (D3) with lower affinity (~1 µM) to TβRII (11). No direct binding of TGM1 to betaglycan (TβRIII) was found (5).

TGM1 also induces a higher maximal level of SMAD transcriptional activation than TGF-β in target cells, such as the MFB-F11 mouse fibroblast reporter cell line, which secretes alkaline phosphatase over a 24-h period in response to TGF-β ligation (5). We confirmed that TGM1 also activates a murine epithelial cell line, NM18-CAGA-dynGFP, containing a SMAD3/4-dependent transcriptional reporter; as with MFB-F11 cells, TGM1 activity in this cell line is fully blocked by a selective small molecule TβRI kinase inhibitor SB505124 but unaffected by neutralizing antibody to mammalian TGF-β (*SI Appendix*, Fig. S1 *A*–*D*). We noted in the MFB-F11 system that the enhanced signaling by TGM1 was lost in truncated versions of TGM1, comprising D1/2/3 or D1/2/3/4, with markedly reduced potency compared to the full-length protein (Fig. 1*B*). In another assay system, using primary murine T cells, the induction of the Foxp3 transcription factor characteristic of TGF-β signaling (17) was likewise attenuated at lower concentrations (Fig. 1*C*).

In parallel, we established that D1/2/3 induced SMAD2 phosphorylation, a key intracellular mediator of the TGF-β pathway, albeit attenuated compared to full-length TGM1; in this 1-h assay, the mammalian and parasite ligands induce comparable signals (Fig. 1*D*); in contrast, no phospho-SMAD2 was observed in cells treated with D4/5 alone. Taken together, these data suggested that D5, either alone or in combination with D4, contributed to the functional potency of the TGM1 protein.

To investigate whether D4/5 drives an independent signal that enhances cell activation, MFB-F11 reporter cells were costimulated with truncated TGM1 (D1/2/3) and D4/5 as separate proteins, over a range of concentrations, but no enhancement was found (Fig. 1*E*). In contrast, when D4/5 was added to full-length TGM1, higher concentrations were able to inhibit, but not abolish, TGF-β signaling (Fig. 1*F* and *SI Appendix*, Fig. S1*E*); D4/5 does not inhibit signaling induced by TGF-β in either NM18-CAGA-dynGFP or primary murine T cells (*SI Appendix*, Fig. S1 *E* and *F*). Hence, D4/5 may bind a coreceptor that consolidates signaling through the canonical TGF-β receptors.

In recent structural studies, key contact residues between TGM1 D3 and TβRII were defined (11). We validated that mutation of Tyr-253 to Ala abolished activity of TGM1 D1/2/3 in reporter fibroblasts, while mutation of a nonbinding site residue (Ile-256) showed no effect (11). However, when the same mutations were made in full-length TGM1, the Y253A mutant regained activity albeit with reduced potency (Fig. 1*G*). Hence, the presence of D4/5 was able to rescue a mutant construct that could only poorly signal in the absence of these accessory domains.

### TGM1 Domains 4 and 5 Bind CD44.

**Fig. 2.**
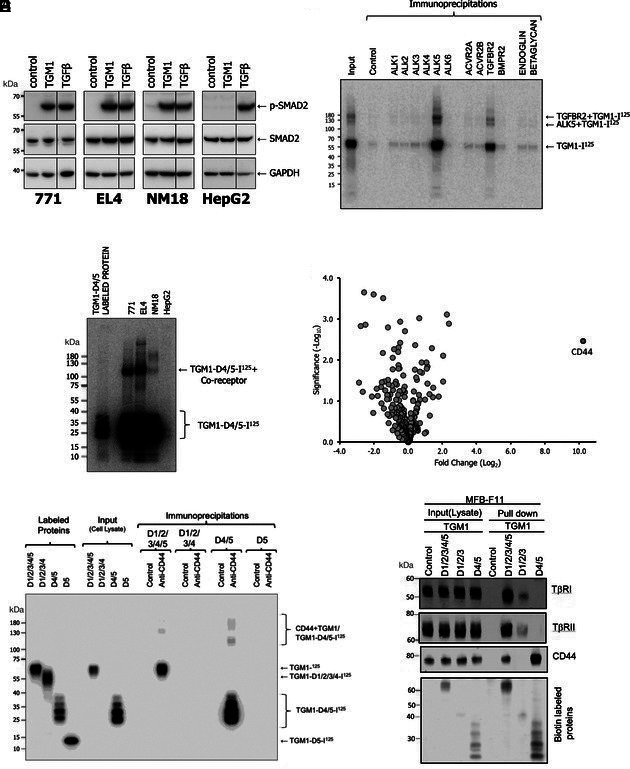
Identification of CD44 as coreceptor for TGM1 signaling. (*A*) Ability of TGM1 (10 ng/mL) and TGF-β (1 ng/mL) to activate SMAD signaling in four cell lines, 771 (murine B cell lymphoma), EL4 (murine thymoma), NM18 (mouse epithelial cells), and HepG2 (human hepatoma), assessed by Western blotting with anti-phospho-Smad2 antibodies after 1 h stimulation in full medium. (*B*) Binding of ^125^I-TGM1 to ALK5 (TβRI) and TβRII, but not other TGF-β/BMP type I and type II family receptors. EL4 cells were incubated with radio-labeled ligand for 3 h on ice, then crosslinked with BS3/DSS (15 min), lysed, and incubated overnight with receptor specific antibodies; finally, complexes were isolated with protein A beads (1 h, 4 °C), separated by SDS-PAGE, and detected by autoradiography. (*C*) Identification of ~80 kDa coreceptor cross-linked to ^125^I-D4/5 on the cell surface of 771, EL, and NM18 cells, but not HepG2 cells, as detected by SDS-PAGE and autoradiography. (*D*) Mass spectrometric identification of CD44 from NM18 cells exposed to biotinylated TGM1 D4/5 and coprecipitated with neutravidin beads. Fold change is calculated compared to control cells that have undergone the same procedure, but without the addition of the TGM1-D4/5-biotin. (*E*) Immunoprecipitation of iodinated full-length TGM1, and D4/5, but not D1/2/3/4 or D5 alone, with anti-CD44 antibody. Intact cells were first incubated with radiolabeled ligands, crosslinked and washed, and thereafter, cell lysates are prepared, as the input into immunoprecipitation protocols. (*F*) Coprecipitation of TGF-β receptor I (ALK5) and TGFβRII from MFB-F11 cells by biotinylated D1/2/3, and CD44 by biotinylated D4/5, with Streptavidin beads and analysis by SDS-PAGE and Western blot with anti-TβRI, anti-TβRII and anti-CD44 as indicated. For panels *A, B, C,* and *F,* Mol.wt markers are shown in kDa on the left-hand side.

To investigate receptor interactions of TGM1, we first surveyed well-characterized cell lines that have previously been studied in the context of TGF-β signaling, to select a suitable substrate from which a D4/5 receptor might be sought. Of four cell lines, three murine cell lines, 771, EL4, and NM18, showed equivalent SMAD2 phosphorylation with TGF-β and TGM1, although the fourth, the human line HepG2, was not activated by TGM1 (Fig. 2*A*). Notably, we did not find a human cell line responsive to TGM1, although all murine cell lines responded (*SI Appendix*, Fig. S2). We selected EL4 for further analysis and then established the binding specificity of TGM1 among the multiple TGF-β family receptors. EL4 cells were incubated with iodinated TGM1 and after crosslinking the cells were lysed. Aliquots of cell lysates were subjected to immunoprecipitation with antibodies to each of six different type I receptors (also termed activin receptor-like kinase (ALK)1-ALK6), four different type II receptors (Activin type IIA and IIB receptors, TGFβRII, and BMP type II receptor), and the type III receptors endoglin and betaglycan. Thereafter, immunoprecipitates were separated on size by SDS-PAGE and ligand-receptor complexes were revealed by autoradiography. The results revealed that only ALK5 (TβRI) and TβRII interacted with TGM1 at levels above background (Fig. 2*B*).

We then used iodinated D4/5 to bind to cells, followed by covalent crosslinking to putative receptors; this technique yielded evidence for a coreceptor/TGM1-D4/5 complex migrating at ~110 kDa, indicating a coreceptor of ~80 kDa, that was present in each of the three responder cell lines, but not in HepG2 cells (Fig. 2*C*). To identify this coreceptor, biotinylated D4/5 was added to NM18 cells and cell lysates were subjected to pull down with neutravidin beads. The precipitates were analyzed by mass spectrometry. This identified CD44 as a highly abundant constituent in the pulldown (Fig. 2*D*). CD44 is highly expressed by many immune system cell populations, binding hyaluronan to mediate adhesion and migration of lymphocytes (18–20). Interestingly, CD44 has previously been reported to associate with TβRI on both murine T cells and human primary and transformed cells (21–23).

Anti-CD44 antibody was then used to perform an immunoprecipitation from cells exposed to iodinated TGM1 constructs lacking either D4 and/or D5; only when both domains are present (either with or without D1/2/3) was there an association with CD44 (Fig. 2*E*). Furthermore, when cells were exposed to biotinylated full-length or truncated TGM1 proteins, and complexes precipitated with streptavidin beads for Western blot analysis, D1/2/3 associated with TβRI and TβRII, while D4/5 linked to CD44 (Fig. 2*F*).

### Biophysical Characterization of TGM1-CD44 Binding.

**Fig. 3.**
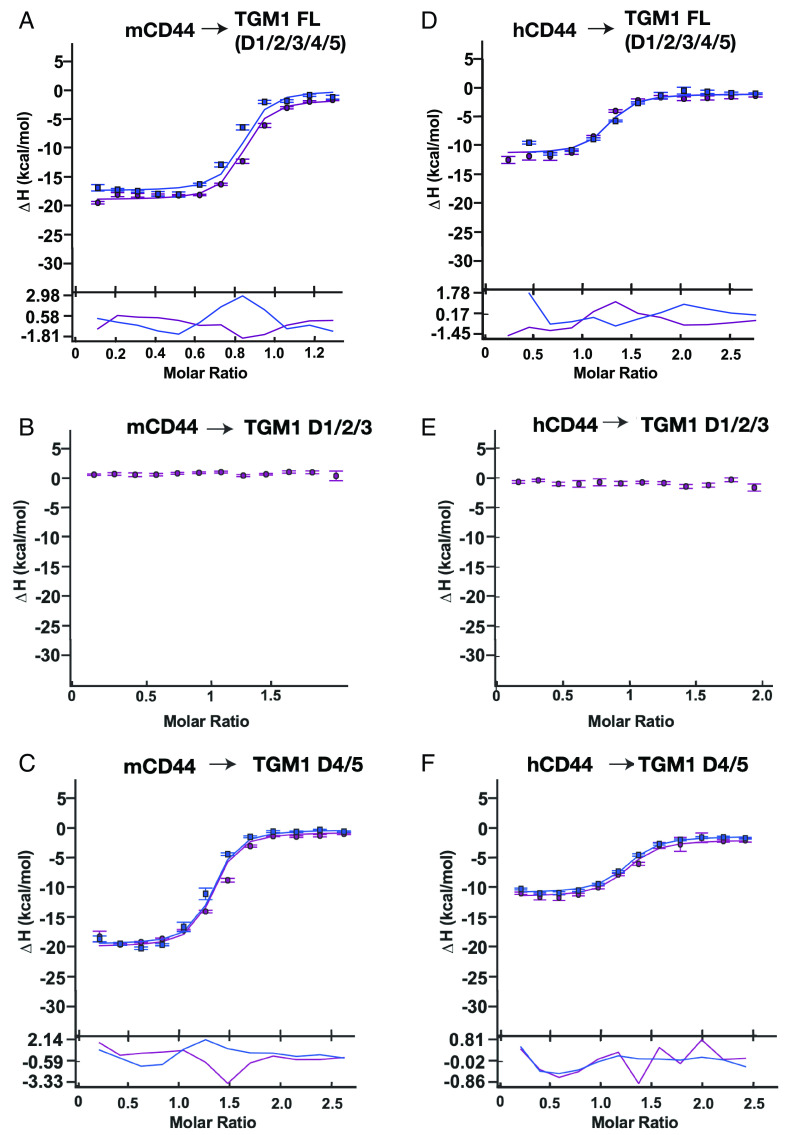
Isothermal calorimetry (ITC) confirmation of TGM1 domains 4/5 binding to murine and human CD44. Integrated heats for each of the sequential injections of mCD44 into TGM1-FL (*A*), TGM1-D1/2/3 (*B*), TGM1-D4/5 (*C*), or hCD44 into TGM1-FL (*D*), TGM1-D1/2/3 (*E*), TGM1-D4/5 (*F*) performed in duplicate (blue and purple shading). Integrated heats (*y* axis) are fit to a 1:1 binding model as a function of CD44:TGM molar ratio (*x* axis) are shown by the smooth continuous lines (blue and purple shading for the duplicate data sets). Error bars indicate bias in the Nitpic program (24) estimation of the integrated heats; the residuals in the fits are shown in the lower portion of each plot.

We next confirmed the interaction between purified TGM1 D4/5 and CD44 by isothermal calorimetry (ITC), which permits binding of proteins in solution to be precisely quantitated to estimate the affinity or dissociation constant (K_D_). As shown in Fig. 3 *A* and *B*, full-length TGM1 binds strongly to murine CD44, but D1/2/3 does not. However, TGM1 D4/5 closely replicates the affinity of the full-length protein (Fig. 3*C*, K_D_ 64 and 62 nM for full-length and D4/5 respectively), confirming that these domains independently bind the CD44 coreceptor. As the human cell line HepG2 did not respond to TGM1, we tested whether TGM1 can also bind to human CD44, which has ~84% amino acid identity with the mouse protein across the exons expressed in all splice variants. Using ITC, we found that the parasite protein does bind the human homolog, dependent on D4/5, albeit with lower affinity than mCD44 (Fig. 3 *D*–*F*, K_D_ 92 and 140 nM for full-length and D4/5). Further data are presented in *SI Appendix*, Fig. S3, and a summary of all interactions observed is provided in Table 1.

**Table 1.** TGM1:CD44 binding as assessed by ITC

Cell	TGM1-FL (D1/2/3/4/5)	TGM1-FL (D1/2/3/4/5)	TGM1-D4/5	TGM1-D4/5	TGM1-D1/2/3	TGM1-D1/2/3
Syringe	mCD44	hCD44	mCD44	hCD44	mCD44	hCD44
Cell concentration (μM)	15	10	12	8	10	10
Syringe concentration (μM)	100	100	90	100	90	100
Temperature ( ${}^{\circ }C$)	35	35	35	35	35	35
K_D_ (nM)	64 (28, 123)^*^^,^^†^	92 (32, 219)^*^^,^^†^	62 (28, 117)^*^^,^^†^	140 (86, 221)^*^^,^^†^	ND^‡^	ND^‡^
Δ H (kcal mol^−1^)	−17.5 (−18.8, −16.3)^*^^,^^†^	−10.3 (−11.8, −9.1)^*^^,^^†^	−19.4 (−20.5, −18.4)^*^^,^^†^	−9.6 (−10.2, −9.0)^*^^,^^†^	ND^‡^	ND^‡^
Δ G (kcal mol^−1^)	−10.1^*^^,^^†^	−9.9^*^^,^^†^	−10.2^*^^,^^†^	−9.7^*^^,^^†^	ND^‡^	ND^‡^
−TΔ S (kcal mol^−1^)	7.3^*^^,^^†^	0.4^*^^,^^†^	9.2^*^^,^^†^	−0.1^*^^,^^†^	ND^‡^	ND^‡^
Stoichiometry (n)	0.8^§^	1.2^§^	1.4^§^	1.2^§^	ND^‡^	ND^‡^

^*^Not determined due to weak signal.

^†^Uncertainty reported as 68.3% CI.

^‡^Global fit of two replicates.

^§^Number of sites determined by incompetent fraction value on sedphat; set to “1” for KD analysis.

### Modulation of CD44 Expression Controls Response to TGM1.

**Fig. 4.**
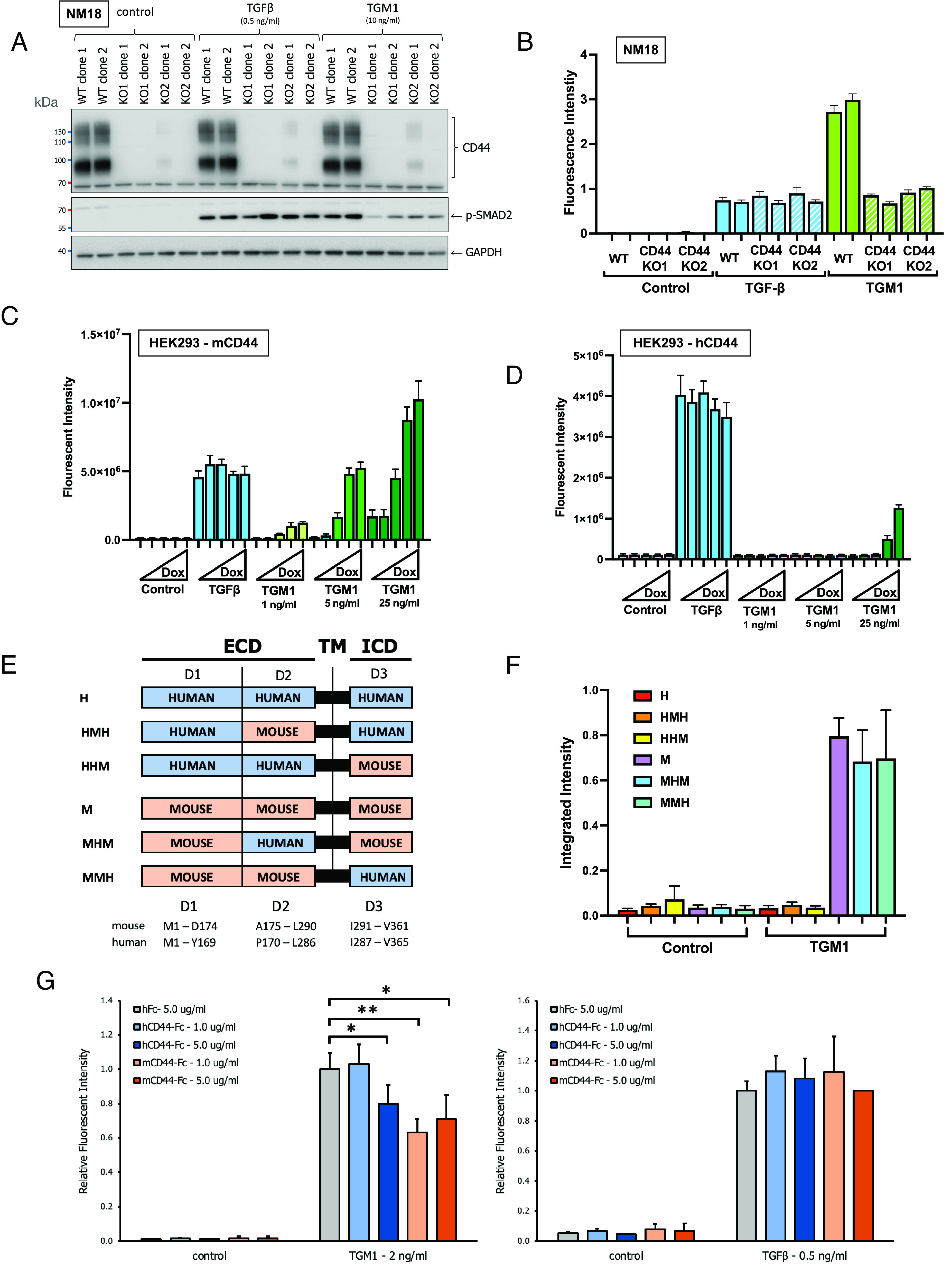
Modulation of CD44 expression controls response to TGM1. (*A*) SMAD2 phosphorylation of murine NM18 cells with intact (Control) or deleted (KO) CD44 gene expression in response to TGF β and TGM1. Cells were stimulated with 0.5 ng/mL TGF-β or 10 ng/mL TGM1 and lysates were analyzed by SDS-PAGE and Western blotting with anti-CD44, anti-pSMAD2 and anti-GAPDH. Two replicate clones of 2 KO guides were tested. Mol.wt markers are shown in kDa on the left-hand side. (*B*) Response of NM18-CAGA-mCHERRYd2 transcriptional reporter cells with CD44 KO induced by 0.5 ng/mL TGF-β and 10 ng/mL TGM1. (*C* and *D*) Response of HEK293-CAGA-mCHERRYd2 transcriptional reporter cells stably transduced with doxycycline-inducible murine (*C*) or human (*D*) CD44, stimulated with TGF-β or the indicated doses of TGM1. (*E*) Schematic of mouse and human CD44 domain swap constructs. ECD, Extracellular Domains; TM, Transmembrane; ICD, Intracellular Domains. The sequences used are mouse CD44, NP_001034240.1; human CD44, NP_001001391.1. Note that the TM domain is fully conserved between mouse and human. (*F*) Response of HEK293T-CAGA-mCherry cells stably transduced with doxycycline-inducible mouse and human CD44 domain constructs. (*G*) Effect of human (hCD44-Fc) or mouse (mCD44-Fc) recombinant CD44-Fc fusion proteins on the response of NIH3T3-CAGA-dynGFP cells to TGM1 (*Left*) or TGFβ (*Right*). Ligands were preincubated with control Fc or CD44-Fc for 30 min before adding to cells and fluorescence measured by IncuCyte with Incucyte software.

To evaluate the role of CD44 in facilitating signaling through the TGF-β receptors, we tested NM18 cells following genetic deletion of the CD44 coding gene, exposing the cells to TGF-β and full-length TGM1. While both parental and CD44-knockout lines have a similar P-SMAD2 signal increase in response to TGF-β stimulation, TGM1-driven SMAD2 signaling was diminished in the CD44 KO lines compared to wild type (Fig. 4*A*). A similar result was seen when using the NM18-CAGA-mCHERRYd2 reporter cells (Fig. 4*B*). Upon stable transduction of human HEK293 cells with a doxycycline-inducible construct for either mouse or human CD44, we found that both showed similar dose-dependent inducible expression of the transduced genes (*SI Appendix*, Fig. S4*A*). CD44 is differentially spliced and the form used for these experiments comprised the first and last 5 exons, without any of the 10 intervening (variable) exons. The stably transduced HEK293 cells expressing murine CD44 were found to gain responsiveness to TGM1 in a doxycycline dose-dependent manner (Fig. 4*C*); however, in this cell type, human CD44 transfection conferred only minimal responsiveness (Fig. 4*D*). To further analyze if there is a functional difference between mouse and human CD44, domain swap constructs were expressed containing N-terminal, central, and/or C-terminal domains of each protein (Fig. 4*E*). Doxycycline-induced expression of these constructs (*SI Appendix*, Fig. S4*B*) revealed that only when the N-terminal domain of mouse CD44 was present, did TGM1 activate cell signaling (Fig. 4*F*). Finally, iodinated TGM1 was used to probe and crosslink HepG2 cells stably transduced with a doxycycline-inducible construct for either mouse or human CD44, and anti-CD44 coimmunoprecipitation showed only interaction with mouse, not human cell surface CD44 (*SI Appendix*, Fig. S4*C*).

We next evaluated whether soluble mouse or human CD44, administered as fusion proteins with their respective Fc domains, could interfere with TGM1 activation; using the NIH3T3-CAGA cell line with a GFP reporter, we found that murine CD44-Fc significantly reduced the response to TGM1, as did human CD44-Fc, albeit to a lesser extent (Fig. 4 *G*, *Left*); neither ligand affected the response of the same cells to TGF-β (Fig. 4 *G*, *Right*).

### TGM1 D4/5 Binds to CD44 on the Cell Surface.

**Fig. 5.**
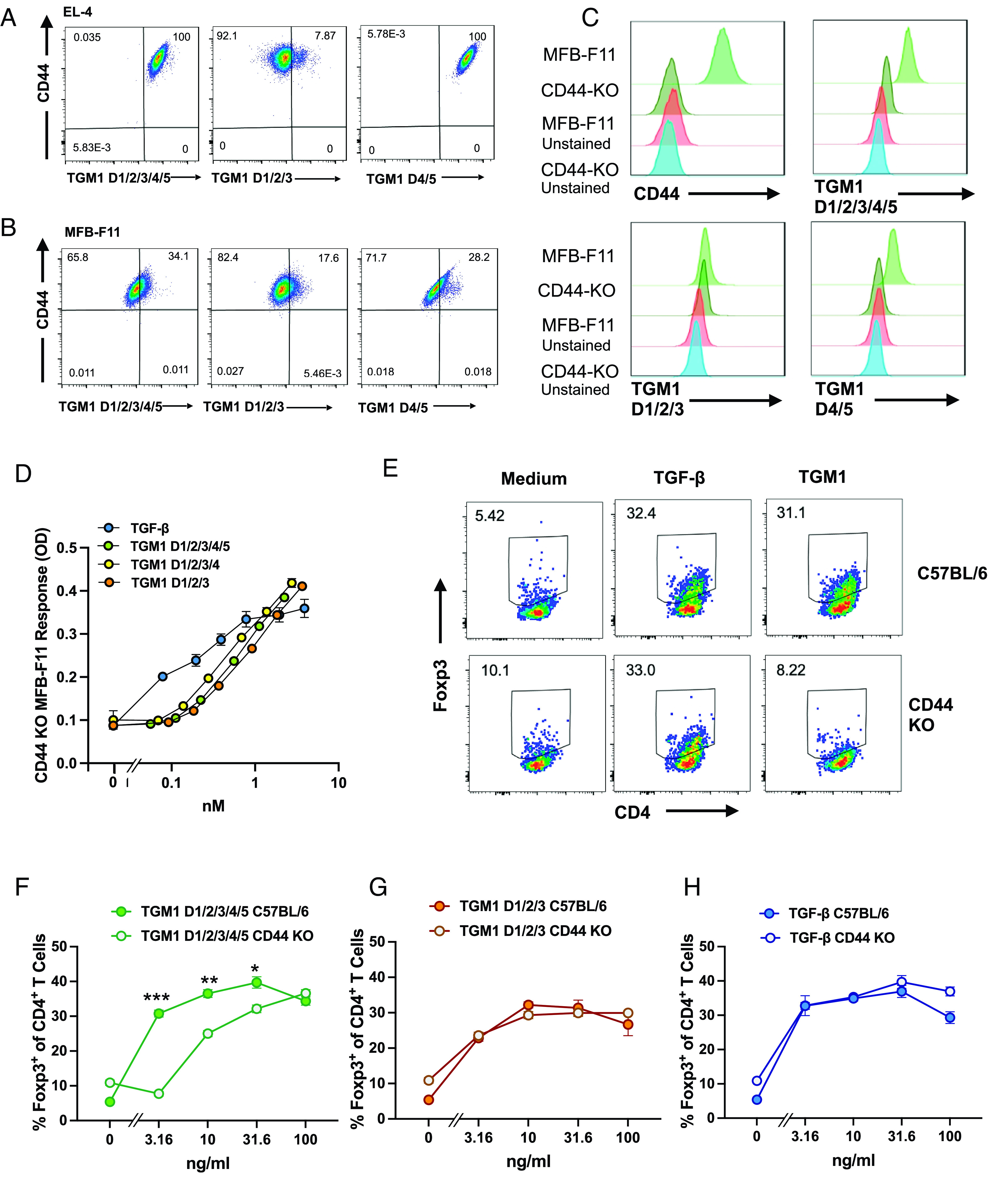
CD44 mediates cell surface binding and enhances Foxp3 induction by TGM1. (*A* and *B*) Costaining of EL-4 (*A*) and MFB-F11 cells (*B*) with anti-CD44 and Alexa-Flour-488 labeled TGM1 full-length (D1/2/3/4/5), D1/2/3, or D4/5 constructs. (*C*) Binding of anti-CD44, or Alexa-Flour-488 labeled TGM1 full-length (D1/2/3/4/5), D1/2/3, or D4/5 constructs to MFB-F11 cells without or with CD44 deletion, with control unstained cells also depicted. (*D*) Deletion of CD44 in MFB-F11 abolishes the enhancement effect of TGM1 D4/5. (*E* and *F*) CD4^+^ splenic T cells from CD44-deficient mice show reduced Foxp3 induction in response to full-length TGM, following coculture with anti-CD3 and IL-2. Statistical comparisons between Foxp3 induction in CD44-sufficient C57BL/6 mice and CD44-deficient animals are shown, *P < 0.05. **P < 0.01, ***P < 0.001 by unpaired *t* test corrected for multiple comparisons. (*G* and *H*) Absence of CD44 on CD4^+^ splenic T cells has no effect on TGF-β signaling stimulated by TGM1 D1/2/3 (*E*), or TGF-β (*F*) under the same conditions.

As CD44 is a surface protein on many cell populations, we used Alexa-Fluor 488-labeled TGM1, D1/2/3, and D4/5 to probe binding by flow cytometry, in combination with anti-CD44 antibody. Strong binding, of 99 to 100% of cells by full-length and D4/5, and by anti-CD44, was found for both EL4 and J774 cells (Fig. 5*A* and *SI Appendix*, Fig. S5*A*) cells. A much lower proportion of cells bound to D1/2/3, while D4/5 bound with a higher intensity than full-length TGM1. Similar results were obtained for the MFB-F11 fibroblast reporter cell line, although to a lower degree, again with enhanced binding by D4/5 (Fig. 5*B*). Binding to MFB-F11 cells by full-length and D1/2/3 TGM1, but not D4/5, was reduced to a limited but significant degree by preincubation with TGF-β (*SI Appendix*, Fig. S5*B*). Combined stimulation with TGM1 and TGF-β was also found to result in a strong pSMAD2 signal irrespective of the presence of CD44, which was required as shown above for potent TGM1 activation (*SI Appendix*, Fig. S5*C*).

### Deletion of CD44 Reduces Binding In Vitro.

We used CRISPR to genetically inactivate CD44 in murine MFB-F11 cells (*SI Appendix*, Fig. S5*D*), and confirmed the ablated expression as measured by binding of anti-CD44 antibody (Fig. 5*C*). The binding of both full-length and TGM1 D4/5 was abolished when tested on CD44 deficient cells (Fig. 5*C*). CD44-knockout and control MFB-F11 cells were then treated with TGM proteins to evaluate the contribution of D4/5; in the knockout cells, full-length TGM1 was much less potent than TGF-β and most notably did not differ from activation by truncated (D1/2/3 and D1/2/3/4) forms (Fig. 5*D*); hence the D4/5-CD44 interaction fully accounts for the added activity of full-length TGM1 when tested on wild-type MFB-F11 cells. When CD44-deficient cells were treated with biotinylated TGM1 for streptavidin-mediated pulldown of associated proteins, both TβRI and TβRII were precipitated to a similar degree as in unmodified cells (*SI Appendix*, Fig. S5*C*).

T cell splenocytes from CD44-deficient C57BL/6 strain mice (25) were then tested for ability to express Foxp3 in response to TGM1 stimulation; compared to wild-type mice fewer Foxp3^+^ cells were induced by TGM1 in the absence of CD44, although activation by TGF-β stimulation was unaffected (Fig. 5*E*). Quantitatively, when full-length TGM1 was used, there was an approximately 10-fold loss in activity with CD44-knockout T cells compared to T cells from wild-type C57BL/6 (Fig. 5*F*); however, when using TGM1 D1/2/3 (Fig. 5*G*), or TGF-β (Fig. 5*H*), there was no differential between the two genotypes of T cell. Hence, the D4/5-CD44 interaction also fully accounts for the enhancement mediated by D4/5 on the T cell population.

## Discussion

The helminth parasite protein, TGM1, faithfully mimics the ability of TGF-β to downregulate immune responses through the canonical TβRI/TβRII receptor/SMAD signaling pathway (5–7). TGM1 comprises 5 homologous domains, of which domains 1/2 and 3 bind independently to TβRI and TβRII, respectively (11). We now show that TGM1 additionally ligates a third binding partner, CD44, through domains 4 and 5. This finding further emphasizes the extraordinary evolutionary adaptation of the CCP gene family from which TGM1 is derived. Our data show that CD44 binding is not essential but increases biological activity by an order of magnitude, amplifying its effect on populations expressing high levels of CD44; the presence of D4/5 also allows the protein to tolerate mutations in D3 that otherwise abolish activity.

Our finding that D4/5 binds strongly to the surface of multiple cell types, including T cells, suggests that D4/5 may serve a targeting role, focussing the effect of TGM1 on cell types most highly expressing CD44. If so, TGM1 may preferentially affect CD44(high) populations such as memory T cells (26), inflammatory granulocytes (27), and dendritic cells (20), as shown in murine studies. In the case of regulatory T cells, CD44 is known to promote expression of the archetypal transcription factor Foxp3 (28), which may contribute to the more potent induction of Tregs by TGM1 compared to TGF-β (6, 7). CD44 is also well represented on a number of other cell types such as intestinal stem cells (29), which have yet to be considered as potential targets for TGM1. Indeed, most cell types express CD44 to some degree (16) indicating that enhancement of TGM1 signaling may either require a threshold level of this coreceptor or that its linkage to the TβRs is regulated in a cell type–specific manner. As the CD44 gene encodes multiple alternatively spliced exons (30), cell-specific expression of splice variants may also impact on interactions with TGM1. A further possibility is that additional coreceptors participate in the TGM1/TβR/CD44 complex, such as EGFR (31), c-Met (32), or PDGFβR (22), an avenue which we are currently exploring.

Hyaluronan, the primary ligand of CD44 (18), has been reported to promote the interaction between CD44 and TβRI, thereby leading to increased SMAD2/3 phosphorylation in metastatic breast cancer cells (21). Moreover, CD44 and TβRI have been found to cluster in a galectin-9-dependent manner in induced murine Treg cells, leading to SMAD3 activation and enhanced stability and function of Treg-mediated suppressor function (23). More broadly, the TGM1-mediated recruitment of CD44 to TβRs may be viewed as a further mimicry and recapitulation of TGF-β signaling, albeit other ligands such as hyaluronan or galectin may be involved. It will be of interest to examine whether TGM1 also requires other molecules, such as galectin-9, to facilitate CD44/TβRI complex formation on the surface of regulatory T cells.

The functional role of CD44 as a coreceptor may reflect one or more alternative possibilities. First, it is likely that CD44 increases the overall avidity of TGM1 for the host cell receptor ensemble, thereby potentiating cellular sensitivity to TGM1 given that its affinity for TβRII alone is tenfold lower than TGF-β (11), yet it signals with comparable potency. Indeed, the ability of D4/5 to override a mutation in D3 that inactivates a truncated protein that only includes D1/2/3 supports this. It is also notable that CD44 has been reported to associate with the intracellular (kinase) domain of TβRI (21), suggesting that the signaling complex may assemble sequentially, initially with CD44, then incrementally interacting with TβRI tethered nearby, and finally recruiting TβRII. This model could explain the slower kinetics of SMAD2/3 phosphorylation by TGM1 compared to TGF-β (7). In this respect, further kinetic studies on live cells would be interesting. Alternatively, it is also possible that CD44 may deliver a secondary signal, independent of TβRI/SMAD signaling. As we were not able to restore full TβR/SMAD mediated potency to truncated TGM1 D1/2/3 by adding D4/5 as a separate protein, this suggests that such downstream SMAD signals would require physical linkage or proximity to the TβR signaling complex, although the mechanism by which this would occur is unclear.

One of the unresolved questions is whether the interactions described here, largely with murine cells, also hold for human cells. While most human cell lines did not respond to TGM1, primary human T cells are fully responsive (5, 6) and preliminary data indicate that D4/5 are required for optimal stimulation. It may be that the lower affinity of TGM1 for human CD44 does not reach a threshold for activation or that in the cells tested, a posttranslational modification takes place that compromises the interaction with TGM1. These points are currently under investigation.

In conclusion, our discovery that the parasite immunomodulator, TGM1, has evolved a third binding specificity by interacting with CD44, demonstrates an exceptional level of complexity in the host–parasite interplay that has been coadapting over long evolutionary time. The new role for D4/5 as a coreceptor binding domain also has important implications for other proteins in the TGM family, of which 10 have so far been identified (13); within these proteins, D4/5 are the most variable indicating that different TGM family members could target different coreceptor “addresses” to preferentially activate specific host cell populations. Learning from these molecular strategies adopted by successful parasites is now providing new and unexpected perspectives on how TGF-β signaling may be enhanced, modulated, or targeted for future therapeutic advantage.

## Materials and Methods

### Mice.

Wild-type C57BL/6 (Envigo, UK), Foxp3-green fluorescent protein (GFP) (33), and CD44-deficient mice (25) (Bar Harbor) were maintained in SPF conditions at the University of Glasgow in accordance with UK standards, local ethical approval, and UK Home Office Project Licence.

### Recombinant Proteins.

Expression was conducted in both *Escherichia coli* and HEK293 cell systems, using His tagged proteins and metal chelating affinity chromatography. *H. polygyrus* TGM1 has been deposited in GenBank as a 422-aa protein with the accession number MG099712. For HEK293 expression, full-length (FL, containing D1/2/3/4/5) TGM1 (aa 19-422, omitting the parasite-encoded signal peptide) was cloned into pSecTag2A (Invitrogen). Truncated forms lacking D4/5 (TGM1-D1/2/3, aa 19-262) or lacking D5 (TGM1 D1/2/3/4, aa 19-243) were similarly constructed. Point mutations at Y253A and I256A were made in both D1-3 and FL TGM1, using gene synthesis. Human and mouse CD44 with NIH NCBI accession numbers XP_047283851 and XP_006498709, respectively, were also expressed in HEK293 cells with coding sequences corresponding to the structurally ordered hyaluronan binding domain (aa 21-169 and 23-174, respectively) cloned downstream of the rat serum albumin peptide, a hexahistidine tag, and a thrombin cleavage sequence (LVPRGS) in pcDNA3.1+ (Invitrogen). Proteins were purified on HiTrap nickel chelating columns (Cytiva) and, as needed, were further polished on Superdex 75 size exclusion columns (Cytiva). The biological activity of TGM1 was validated in the CAGA-dynGFP transcriptional reporter (34), confirming that TGM1 signaling, like TGF-β, could be totally ablated by SB505124 (35) (*SI Appendix*, Fig. S1*A*) but was unaffected by anti-TGF-β neutralizing antibody (*SI Appendix*, Fig. S1*B*). Recombinant Human CD44 Fc Chimera protein (art# 3660-CD-050) and Recombinant Mouse CD44 Fc Chimera protein (art# 6127-CD-050) were purchased from R&D system (Minneapolis, USA).

### ITC.

ITC datasets were generated using a Microcal PEAQ-ITC instrument (Malvern Instruments). All experiments with CD44 were performed in 25 mM sodium phosphate, 50 mM NaCl, pH 6.0 at a temperature of 35 °C. Proteins included in the syringe and sample cell were dialyzed against ITC buffer and concentrated as necessary prior to being loaded into either the syringe or sample cell. Protein concentrations in the cell and syringe are indicated in Table 1. CD44 experiments were carried out with 19 2.0-µL injections with an injection duration of 4 s, a spacing of 150 s, and a reference power of 10. Integration and data fitting were performed using the programs Nitpic (24), Sedphat (36, 37), and GUSSI (38).

### Cell Lines.

The following cell lines, 293T, NIH3T3, NM18 (a subline of the mouse mammary gland epithelial cell line NMuMG, ref. (39), MFB-F11 (40), and HepG2 were maintained in DMEM (41966-052, Gibco) supplemented with 10% fetal bovine serum (FBS) (S1810-500, Biowest) and penicillin/streptomycin. EL4 and 771 cells were maintained in IMDM (31980-038, Gibco) supplemented with 10% FBS and penicillin/streptomycin. Cells were frequently tested for absence of *Mycoplasma* and human cell lines were authenticated by Short Tandem Repeat profiling.

### Reporter Cell Assays.

MFB-F11 cell reporter assays, which drive alkaline phosphatase expression, were conducted as originally described (40). Briefly, confluent cells were detached with trypsin, and resuspended in DMEM with 2.5% FCS, 100 U/mL of penicillin, 100 µg/mL of streptomycin and 2 mM L-glutamine at a concentration of 8 × 10^5^ cells/mL. In 50 µL, 4 × 10^4^ cells were added to each well of a 96-well flat-bottomed plate. Purified proteins were then added to each well in a volume of up to 50 µL and incubated for 24 h at 37 °C. Subsequently, 20 µL of the supernatant was aspirated from each well, added to an ELISA plate (Nalge Nunc International, USA) with 180 µL of reconstituted Sigma FastTM p-nitrophenyl phosphate substrate, and incubated at room temperature in the dark overnight. Plates were read at 405 nm on an Emax precision microplate reader (Molecular Devices).

### Plasmids.

The CAGA-dynGFP lentiviral vector was used as previously described (34). The pLV-CAGA-mCHERRYd2-SV40-BSDr was generated from the pLV-CAGA-dynGFP, by replacing dynGFP with mCHERRYd2 and by replacing the PGK-PUROr cassette by a SV40-BSDr cassette. Mouse CD44s (from EL4 cDNA), and human CD44s (a kind gift from Mitsuyasu Kato, University of Tsukuba, Tsukuba, Japan) were cloned using PCR into the pENTR1A vector (Invitrogen). Using the GATEWAY system (Invitrogen) a doxycycline-inducible lentiviral construct was generated in the pCW57.1 vector (a gift from David Root; Addgene plasmid # 41393). Two different knockout guides against mouse CD44 were designed using the ChopChop webtool (https://chopchop.cbu.uib.no/) (Target sequences; guide1: AGCACGCCATGGACAAGTTTTGG, guide2: CACTCACCGATCTGCTGATGTGG). The guides were cloned in the pLENTI-CRISPR-V2 plasmid (pLENTI-CRISPR-V2 was a gift from Feng Zhang, Addgene plasmid # 52961) (41).

### Transcriptional Florescent Protein–Based Reporter Assay.

Cells containing either the CAGA-dynGFP or the CAGA-mCHERRYd2 reporter were seeded in 96-well plates. Cells were stimulated the next day (in full media) and were placed in the IncuCyte S3 live-cell imaging analysis system (Sartoris). The cells were subsequently imaged every 3 h for a period of 48 h. Fluorescence intensity was analyzed using the IncuCyte software.

### Lentiviral Cell Transfection, Knockdown, and Selection.

To make stable cell lines, cells were transduced with lentivirus that was made in 293T cells. In brief, 293T cells were transfected using helper plasmids and the lentiviral plasmid, and on the following day, the transfection media was refreshed. On the second day, the conditioned media was collected and filtered to obtain a cell-free solution. Next, cells were incubated overnight with the filtered conditioned media; 48 h later, cells were selected using an appropriate amount of antibiotic. After selection for the knockouts, single-cell clones were made.

### CRISPR-Mediated Knockout of CD44 in MFB-F11 Cells.

For deletion of CD44 expression in MFB-F11 fibroblast cell lines, a CRISPR strategy was used (42), with guides 1 (AGCACGCCATGGACAAGTTTTGG), and 2 (CACTCACCGATCTGCTGATGTGG) cloned in pSpCas9(BB)-2A-GFP (a kind gift from Jamie Whitelaw, CRUK Beatson institute, Glasgow, Addgene plasmid #48138); 2.5 µg empty and CD44 guide RNA containing pSpCas9(BB)-2A-GFP plasmids were transfected in MFBF-11 cells using lipofectamine 2000 (Invitrogen) in a six-well plate. Twenty-four hours post transfection GFP positive single cells were sorted in 96-well plates. Single-cell clones were screened by Western blotting after approximately 2 wk.

### Iodination and Crosslinking.

Purified recombinant proteins were labeled by iodination, biotinylation, and fluorescent coupling. Iodination of TGM1 proteins was performed by the chloramine T method, and cells were subsequently affinity-labeled with the radioactive ligand as previously described (43, 44). In short, cells were incubated on ice for 3 h with the radioactive protein. After incubation, the cells were washed and crosslinking was performed using 0.27 µM disuccinimidyl suberate (DSS, Pierce, 21555) and 0.07 µM bis(sulfosuccinimidyl) suberate (BS3, Pierce, 21580) for 15 min. Cells were washed, scraped, and lysed. Lysates were incubated with antibodies overnight (4 °C) and were precipitated using protein A Sepharose (Amersham, 17-0963-03). Samples were washed, boiled in SDS sample buffer, and subjected to SDS-PAGE. Gels were dried, exposed to a phorphor screen (FUJIFILM, BAS-SR2040), and then imaged using the Typhoon (Amersham).

### Biotinylation and Pulldown.

Ten µg of TGM-1 full length (D1/2/3/4/5), D1/2/3, and D4/5 was incubated with EZ-Link™ Sulfo-NHS-LC-Biotin (Pierce, 21335) for 30 min at RT°, and the reaction was stopped by the addition of 50 mM Tris-HCl pH 7.4. The reaction was purified using a G-25 column (GE health, 28922529). For the pulldown, cells were washed with PBS and then incubated with biotinylated protein(s) for 3 h on ice. After incubation, the plates were washed 3× with PBS (Fresenius Kabi) and the cells were harvested in 1× Cell Lysis Buffer (Cell Signaling Technologies, 9803). After spinning, the supernatant was incubated with Neutravidin beads (Pierce, 29201) for 1 h at 4 °C (rotating). Beads were washed 4× using lysis buffer and 3× using 50 mM ammonium bicarbonate (Sigma-Aldrich, 09830), with fresh LoBind tubes used for each wash (Eppendorf, 0030 108.116). The beads were resuspended in 250 µL of 50 mM ammonium bicarbonate with 250 ng of mass spec grade trypsin (Promega V5113), incubated overnight at 37 °C (with agitation), after which peptides were recovered from the beads with a prewashed 0.4-µm filter (Ultrafree MC HV, Millipore, UFC30HV00) and subjected to mass spectrometry analysis.

### Western Blotting.

Cell lysates and pull-down samples were analyzed on 4 to 12% bis-tris SDS-PAGE gels and transferred onto nitrocellulose membrane using iBlot2 (Invitrogen, IB21001). Membranes were treated in 5% nonfat milk blocking solution for 1 h, incubated with primary antibody (1:1,000 in 5% BSA containing TBST) overnight at 4 °C, and washed 3× (5 min) using 1× TBST. Fluorescently conjugated secondary antibody (1:10,000 in 5% BSA containing TBST) was used to detect the protein bands by Odyssey CLx Imaging System (LI-COR Biosciences).

### Antibodies for Western Blotting.

The following antibodies were used in Western blot experiments: anti-CD44 (clone epr18668, Abcam, ab189524), anti-GAPDH (clone 6C5, Millipore, MAB374), anti-GFP (D5.1, Cell Signaling Technology 2956), anti-SMAD2/3 (BD Biosciences 610842), anti-Phospho-SMAD2 (45), anti-TβRI (EPR20923-13 Abcam, ab235578), anti-TβRII (EPR24349-124, Abcam, ab259360), and anti-α Tubulin (DM1A, Abcam, ab7291).

### Mass Spectrometry.

Peptides were desalted using StageTips (46) and measured in an Orbitrap Fusion LUMOS (Thermo Fisher Scientific) trybrid mass spectrometer coupled to EASY-nLC 1200 system (Proxeon, Odense, Denmark). Three technical repeats were performed injecting 2%, 10%, and 50% of the sample, respectively. Digested peptides were separated using a 50-cm-long fused silica emitter (FS360-75-15-N-5-C50, New Objective, Massachusetts, US) in-house packed with 1.9 µm C18-AQ beads (Reprospher-DE, Pur, Maisch, Ammerburch-Entringen, Germany) and heated to 50 °C in a Column Oven for ESI/Nano Spray (Sonation, Germany). Peptides were separated by liquid chromatography using a gradient from 2 to 32% acetonitrile with 0.1% formic acid for 30 min followed by column reconditioning for 22 min. A Lock Mass of 445.12003 (polysiloxane) was used for internal calibration. Data were acquired in a Data Dependent Acquisition (DDA) mode with a TopSpeed method with a cycle time of 3 s with a scan range of 400 to 1,500 m/z and resolutions of 120,000 and 30,000 for MS1 and MS2, respectively, using in both cases the Orbitrap as detector. For MS2, an isolation window of 1.2 m/z and an HCD collision energy of 32% was applied. Precursors with a charge of 1 and higher than 6 were excluded from triggering MS2 as well as previously analyzed precursors with a dynamic exclusion window of 10 s.

### Mass Spectrometry Data Analysis.

Mass spectrometry data were analyzed using MaxQuant v1.6.14.0 (47) with the following modifications: Maximum missed cleavages by trypsin was set to 2. Searches were performed against an in silico digested database from the mouse proteome including isoforms and canonical proteins (Uniprot, 22nd April 2021). Oxidation (M), Acetyl (Protein N- term), were set as variable modifications with a maximum of 3. Carbamidomethyl (C) was disabled as fixed modification. Label-free quantification was activated not enabling Fast LFQ. The match between runs feature was activated with default parameters.

MaxQuant output data were further processed in the Perseus Computational Platform v1.6.14.0 (48). LFQ intensity values were log2 transformed, and potential contaminants and proteins identified by site only or reverse peptide were removed. Samples were grouped in experimental categories and proteins not identified in 3 out of 3 replicates in at least one group were also removed. Missing values were imputed using normally distributed values with a 2.1 downshift (log2) and a randomized 0.1 width (log2) considering whole matrix values. Two-sided t tests were performed to compare groups. Analyzed data were exported from Perseus and further processed in Microsoft Excel 365 for comprehensive visualization.

### T cell Assays.

Primary mouse naive T cells were prepared from spleens by passing through a 70-µm cell strainer; contaminating red blood cells (RBC) were removed by resuspending the cells in RBC lysis buffer (Sigma) for 2 min at room temperature. Cells were then washed and resuspended in media made up of RPMI1640, supplemented with 2 mM L-glutamine, 10% heat-inactivated FBS, nonessential amino acids, 100 U mL^−1^ of penicillin, and 100 µg mL^−1^ of streptomycin (all purchased from Thermo Fisher). The cell suspension was then enriched for CD4^+^ T cells using the AutoMACS system (Miltenyi Biotec, Bergisch Gladbach, Germany) and the mouse CD4^+^ T cell isolation kit with “depletes” setting as per the manufacturer’s instructions. Cells were cultured at a concentration of 2 × 10^6^ mL^−1^ in flat-bottomed 96-well plates (Corning, New York, USA) coated with 10 µg mL^−1^ α-CD3 (Life Technologies, Carlsbad, California, USA) for flow analysis or 7.5 × 10^5^ cells in 24-well plate (Life Technologies) for cell sorting experiments. Additional IL-2 (Miltenyi Biotec Biotec) was added at a final concentration of 400 U mL^−1^, and cytokines/proteins were added at the concentrations given for individual experiments. Cultures were incubated at 37 °C with 5% CO_2_ for at least 72 h before being analyzed for flow cytometry for Foxp3 expression.

### Labeling of TGM1 with Alexa Fluor 488.

TGM1 FL (D1/2/3/4/5), D1/2/3, and D4/5 were labeled with Alexa Fluor 488 dye using Alexa Fluor™ 594 Microscale Protein Labeling Kit (Invitrogen™, A30006) as per the manufacturer’s manual. Briefly, 50 µg (~1 mg/mL) protein was mixed with Alexa Fluor 594 dye and 1 M sodium bicarbonate at 1/10th of the reaction volume concentration and incubated at room temperature for 15 min. Unlabeled dye was removed from reaction mixture using the desalting column supplied in the labeling kit. Protein concentrations were calculated using a Nanodrop spectrophotometer (Thermo Scientific).

### Flow Cytometry.

Cells were washed in PBS and stained for viability using FVS510 (BD Biosciences) which was diluted 1:1,000 in PBS and 100 µL was added to each sample of cells and left in the dark at room temperature for 15 min and then washed twice in FACs buffer (PBS containing 0.5% BSA, 5 µM EDTA and 0.5% sodium azide). To reduce nonspecific binding, samples were incubated with 50 µL of polyclonal rat IgG (Sigma) (diluted 1:50 in FACs buffer) for 15 min on ice and protected from light, then washed once in FACs buffer by spinning at 400 *g* for 5 min and removing supernatant. Samples were then stained for flow cytometry by adding 50 µL of anti-CD44-FITC (clone IM7 at 1/200; Biolegend) in Brilliant Staining Buffer (BD Biosciences). Staining with TGMs used Alexa fluor-labeled proteins made as described above. TGMs labeled with AF488 and AF594 were used at a final concentration of 5 μg/mL. Samples that were also stained for intracellular antigens were fixed and permeabilized using Foxp3 Transcription Factor Buffer kit (Invitrogen, USA) and stained with anti-Foxp3-ef450 (clone FJK-16s, eBioscience, San Diego, California, USA, 1/100) in perm/wash buffer. Samples were then washed prior to analysis on a BD Celesta flow cytometer (BD Biosciences).

### Statistical Analysis.

Statistical analyses used one-way ANOVA or unpaired *t* tests, with correction for multiple comparisons, with Prism 9.

## Supplementary Material

Appendix 01 (PDF)

## Data Availability

The mass spectrometry proteomics data have been deposited to the ProteomeXchange Consortium via the PRIDE partner repository (49) with the dataset identifier PXD PXD037799.
