# Supplementary material for: CD44 acts as a coreceptor for cell-specific enhancement of signaling and regulatory T cell induction by TGM1, a parasite TGF-β mimic
Source: Proc Natl Acad Sci U S A. 2023 Aug 17;120(34):e2302370120. doi: 10.1073/pnas.2302370120 (PMC10450677; doi:10.1073/pnas.2302370120)
Supplement: Supplementary file 1 — Appendix 01 (PDF) [file pnas.2302370120.sapp.pdf]

PNAS

Supporting Information for:

CD44 acts as a coreceptor for cell-specific enhancement of signaling and regulatory T cell induction by TGM1, a parasite TGF-β mimic

Maarten van Dinther, Kyle T. Cunningham, Shashi Prakash Singh, Madeleine P. J. White, Tiffany Campion, Claire Ciancia, Peter A. van Veelen, Arnoud H. de Ru, Román González-Prieto, Ananya Mukundan, Chang-Hyeock Byeon, Sophia R. Staggers, Cynthia S. Hinck, Andrew P. Hinck, Peter ten Dijke, and Rick M. Maizels*

*Rick Maizels
Email: Rick.Maizels@glasgow.ac.uk

This PDF file includes:

Figures S1 to S5
Figure Legends S1 to S5

Van Dinther *et al.*, Suppl. Fig. 1

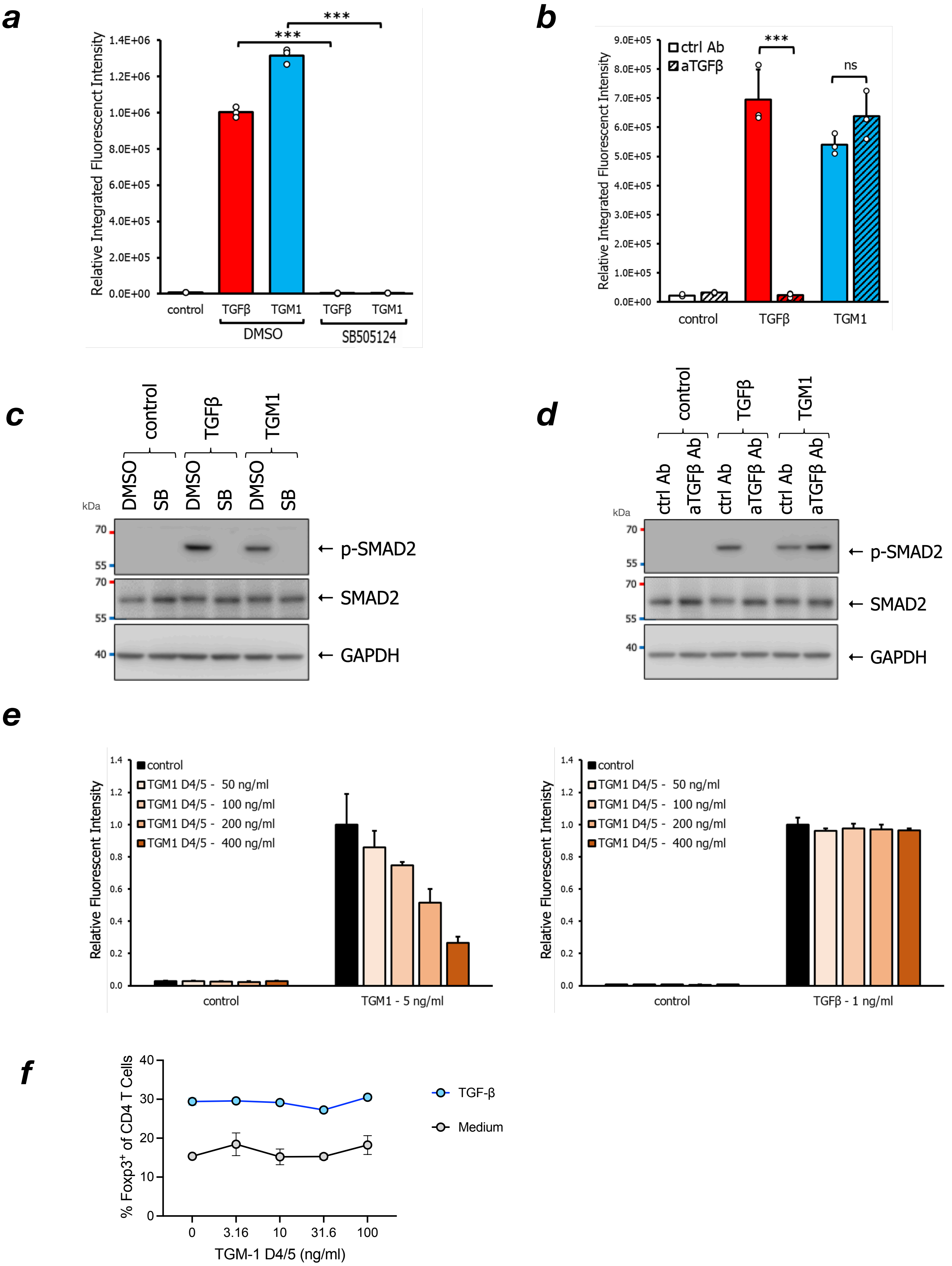

Van Dinther *et al.*, Suppl. Fig. 2

a

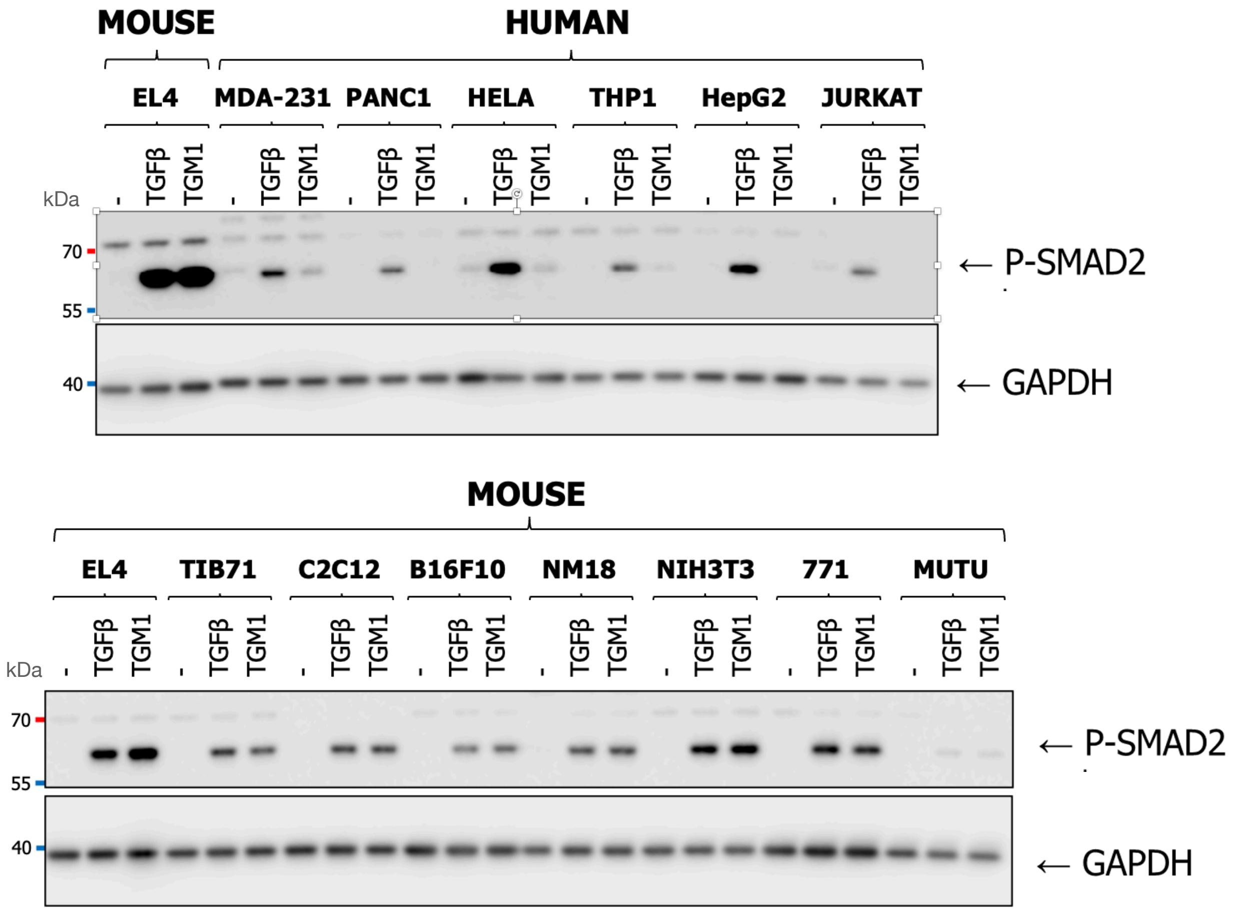

b

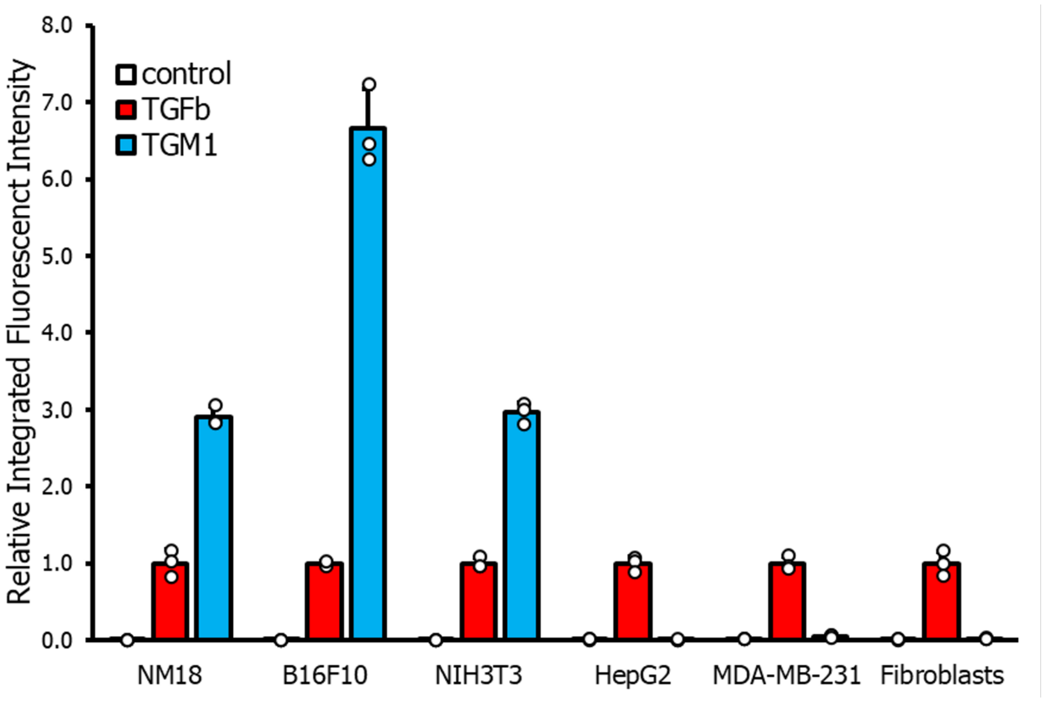

Van Dinther *et al.*, Suppl Fig. 3

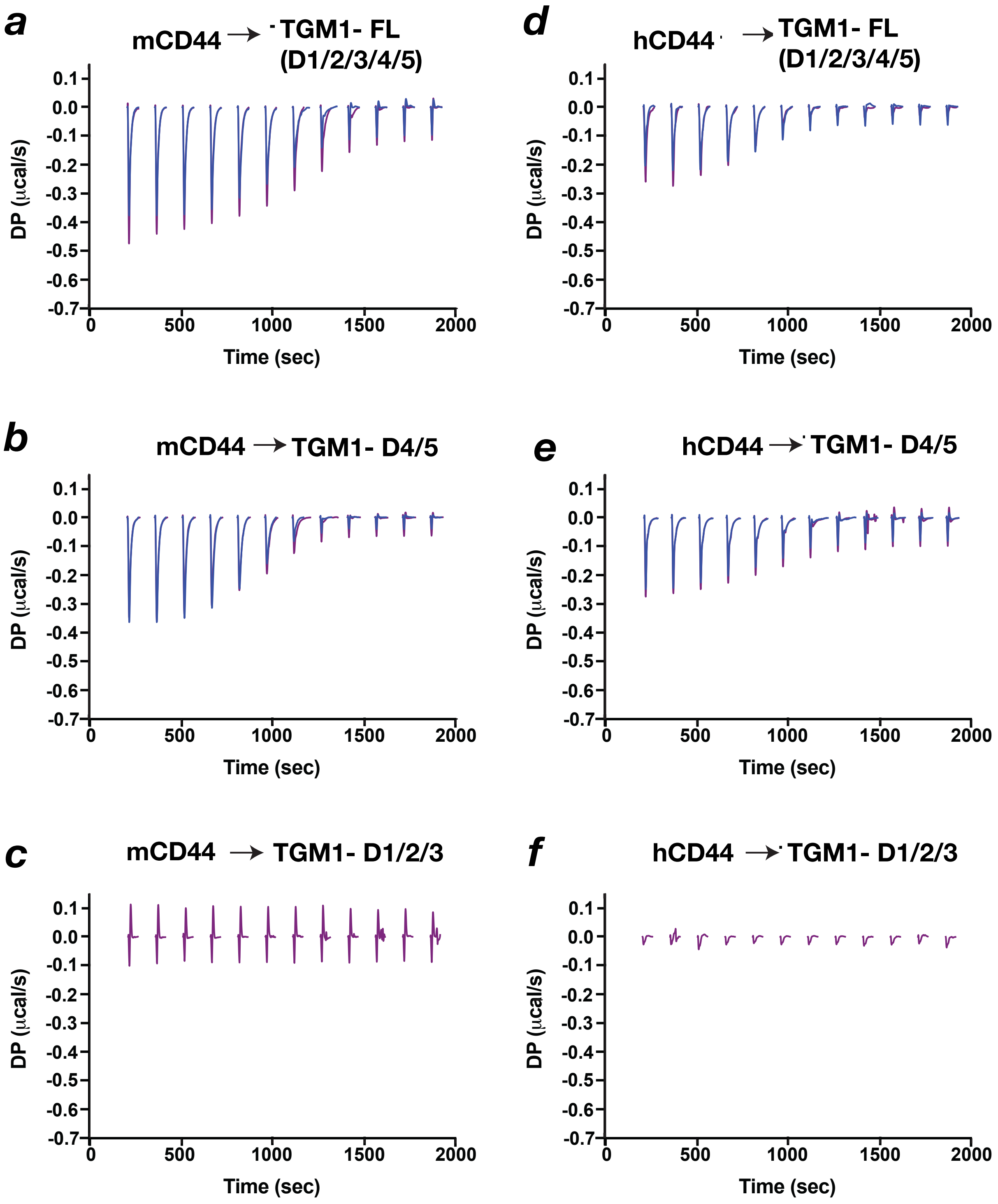

Van Dinther *et al.*, Suppl. Fig. 4

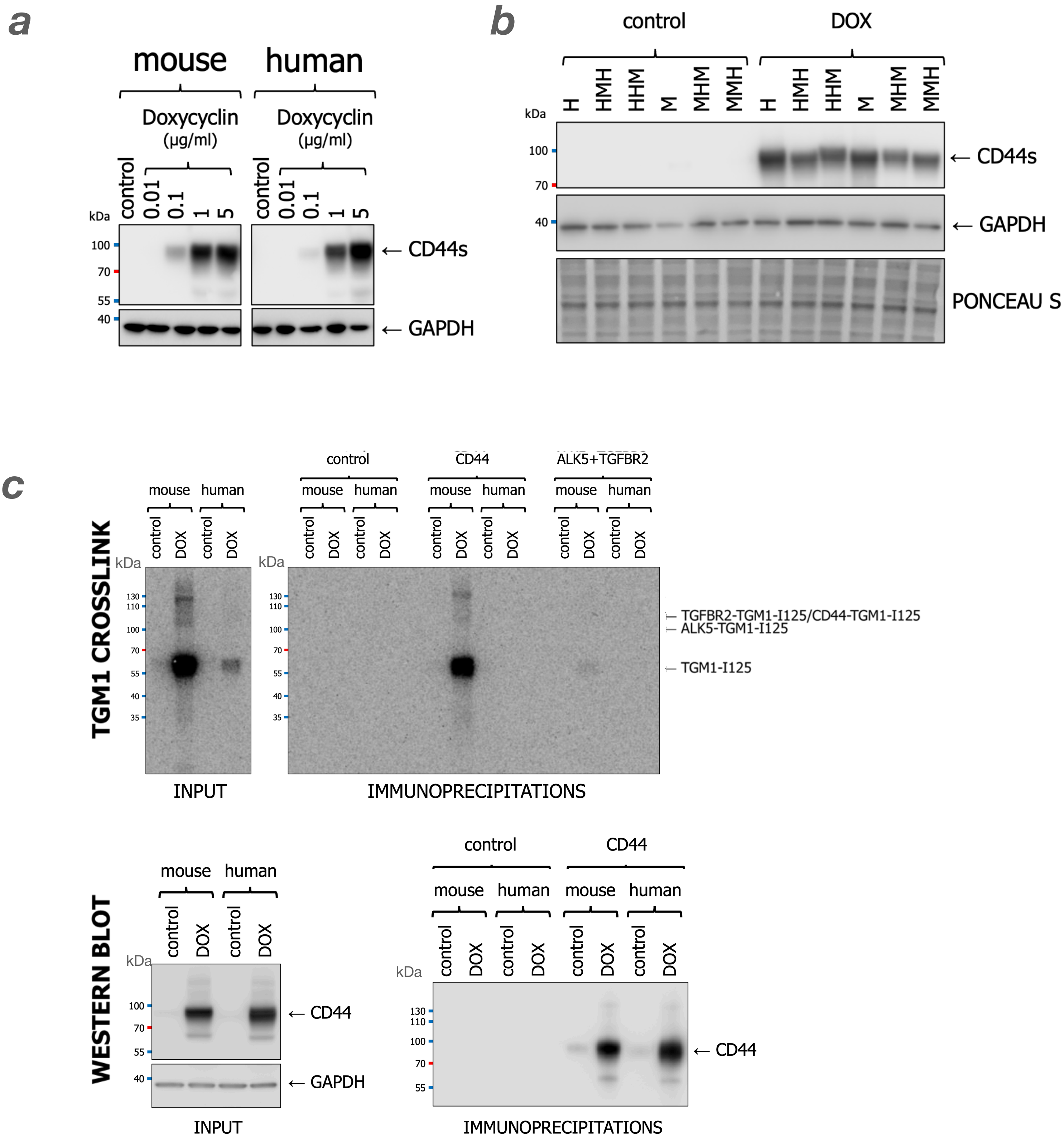

Van Dinther *et al.*, Suppl Fig. 5

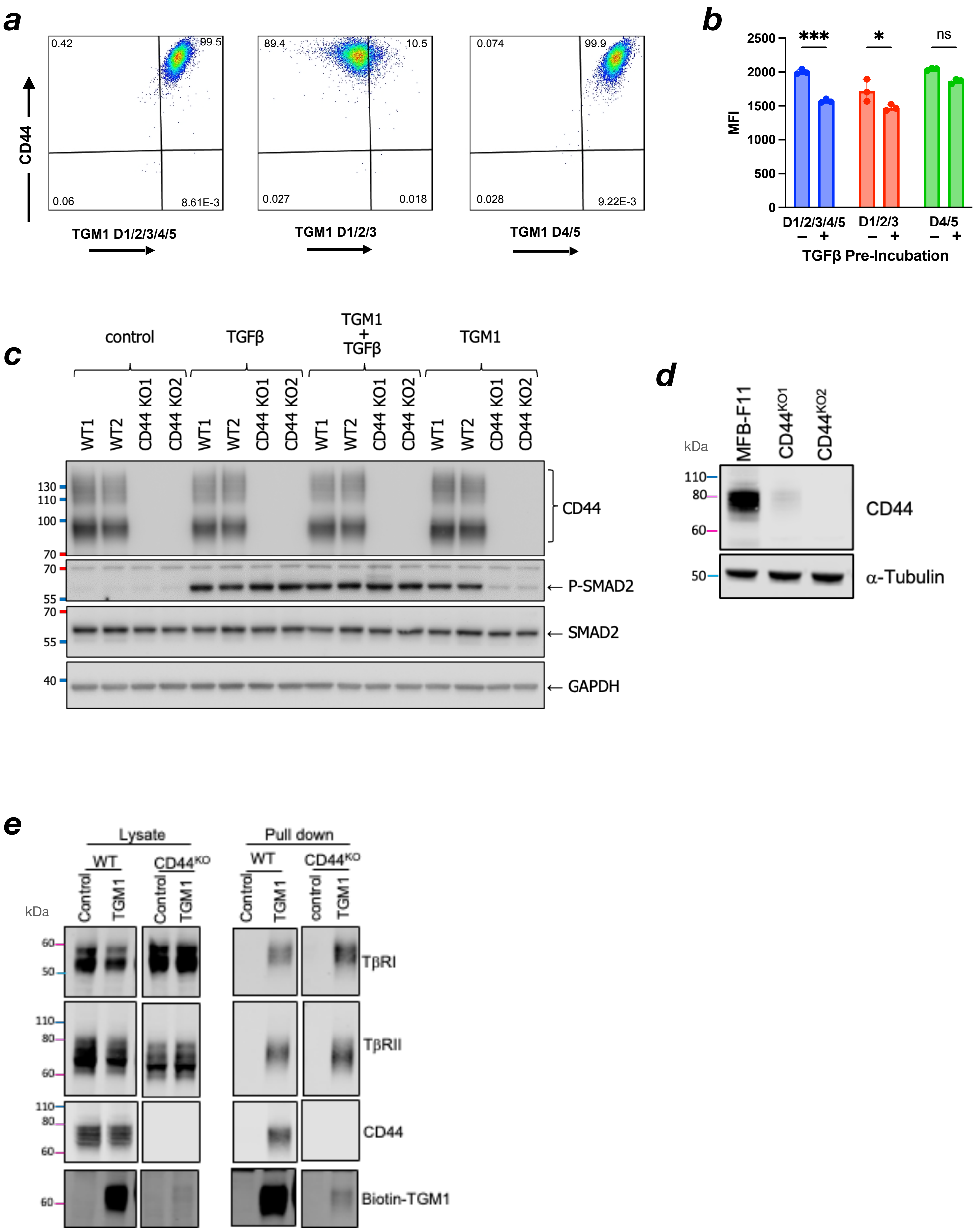

Supplementary Figure Legends

Suppl. Fig 1 : Validation of biological activity of recombinant TGM1, and lack of inhibition of TGF-β activity by D4/5 of TGM1

a TGM1 and TGF-β both activate NM18-CAGA-dynGFP reporter cells in a manner fully inhibitable by 10 μM SB505124.

b TGF-β, but not TGM1, is inhibited by 10 μg/ml anti-TGF-β antibody in NM18-CAGA-dynGFP reporter cells, preincubated with antibody for 30 mins.

c,d As (a, b) with NM18 cells treated as above and analysed by Western blot with anti-SMAD antibodies. Mol.wt markers are shown in kDa on the left-hand side.

e. TGM1, but not TGF-β, activation o*f* NM18-CAGA-dynGFP reporter cells is partially inhibited by addition of TGM1 D4/5 *in vitro.*

f. Addition of TGM1 D4/5 to murine T cell cultures does not inhibit the induction of Foxp3 by TGF-β.

Suppl. Fig 2 : Mouse cell lines, but not human cell lines, are activated by TGM1

a Each of the indicated cell lines were incubated in control medium (-) or with 1 ng/ml TGF-β or 10 ng/ml TGM1 for one hour, and lysates then probed by Western Blot with anti-phospho-SMAD antibody, or anti-GAPDH loading control. Mol.wt markers are shown in kDa on the left-hand side.

b. Responses of CAGA-dynGF-transfected mouse and human cell lines to 1 ng/ml TGF-β or 10 ng/ml TGM1for one hour.

Suppl. Fig 3 : TGM1 binding to mouse and human CD44 by ITC. A-F.

Thermograms for the injection of mCD44 into TGM1-FL (*a*), TGM1-D45 (*b*), TGM1-D123 (*c*) or the injection of hCD44 into TGM1-FL (*d*), TGM1-D45 (*e*), TGM1-D123 (*f*).

Suppl. Fig 4 : Knockout or Transfection with mouse or human CD44 in cell lines

a CD44 expression in stably transduced 293T cells with doxycycline-inducible mouse or human CD44, assessed by Western blot with antibodies to CD44 or GAPDH control. Mol.wt markers are shown in kDa on the left-hand side.

b Expression of all domain swap constructs of CD44 with human (H) or murine (M) N-terminal distal extracellular, central proximal extracellular and C-terminal cytoplasmic

domains, as confirmed by Western blot. Mol.wt markers are shown in kDa on the left-hand side.

c Upper Panel : Immunoprecipitation following cross-linking with ^{125}I-labeled TGM1 performed on HepG2 cells stably transduced with doxycycline-inducible mouse or human CD44, using anti-CD44 (IM7 antibody) or anti-ALK5+TβRII;

Lower Panel: confirmation by Western blot of doxycycline-induced CD44 expression in both mouse and human transfected cells.

Mol.wt markers are shown in kDa on the left-hand side.

Suppl. Fig 5. Staining and Knockout of CD44 in murine cells

a CD44 and TGM1 staining of J774 cells by flow cytometry.

b TGF-β inhibition of TGM1 staining of MFB-F11 cells, compared with full length (D1/2/3/4/5) ligand, or truncated constructs D1/2/3. Or D4/5. * $p<0.05$, ** $p<0.01$.

c P-SMAD responses of NM18 wild-type (WT) or CD44-KO cells stimulated individually with TGF-β (0.25 ng/ml), TGM1 (2.5 ng/ml) or both ligands simultaneously.

d. CRISPR based knockout of CD44 in of MFB-F11 cells confirmed by Western blot. Mol.wt markers are shown in kDa on the left-hand side.

e Streptavidin pull down using biotin TGM1 with MFB-F11 and CD44 knockout cells. Mol.wt markers are shown in kDa on the left-hand side.
